# Supplementary material for: Oncostatin M-Enriched Small Extracellular Vesicles Derived from Mesenchymal Stem Cells Prevent Isoproterenol-Induced Fibrosis and Enhance Angiogenesis
Source: Int J Mol Sci. 2023 Mar 30;24(7):6467. doi: 10.3390/ijms24076467 (PMC10095085; doi:10.3390/ijms24076467)
Supplement: Supplementary file 1 [file ijms-24-06467-s001.zip › ijms-2321356-supplementary.pdf]

Tejedor-Gascón, et al. Supplemental Figure S1.

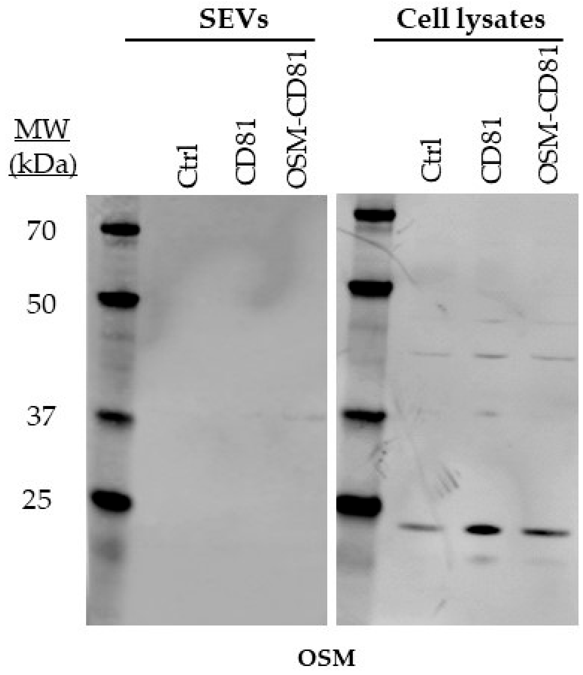

Supplementary Figure S1. OSM protein detection on SEVs and cell lysates samples from Expi293F transiently transfected using the optimized Expi293F expression system. Cells were transiently transfected with lentiviral plasmids for CD81TM and OSM-CD81TM overexpression, and OSM protein was evaluated by Western Blot. SEVs and protein samples from non-transfected cells were used as control (Ctrl). Fusion of one protein with another is represented with a dash (-). Representative Western Blot membranes for each experimental condition is shown.

Tejedor-Gascón, et al. Supplemental Figure S2.

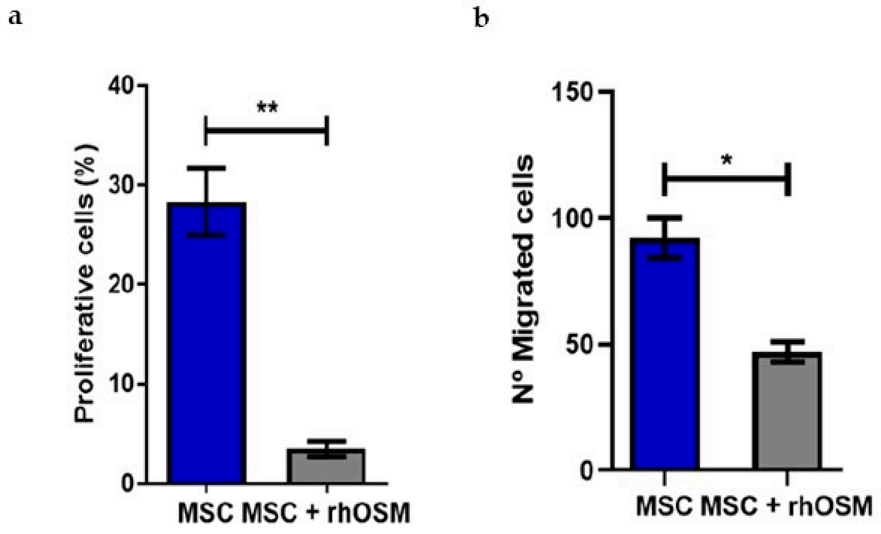

Supplementary Figure S2. Decrease in proliferation and migration of human MSC primary cultures after treatment with recombinant human oncostatin M (rhOSM). **(a)** Percentage of proliferative cells untreated (MSC) or treated with rhOSM (MSC+rhOSM, dose: 10 ng/mL) after BrdU assay. **(b)** Number of migrated cells in basal conditions (MSC) and after rhOSM treatment (MSC+rhOSM, dose: 10 ng/mL) measured by Transwell assay. Two independent experiments were included. Unpaired t-test was used to compare the means. Asterisks represent statistically significant differences (*$p<0.05$; **$p<0.001$). Mean±SEM for each data set is represented.

Tejedor-Gascón *et al*. Supplemental Figure S3.

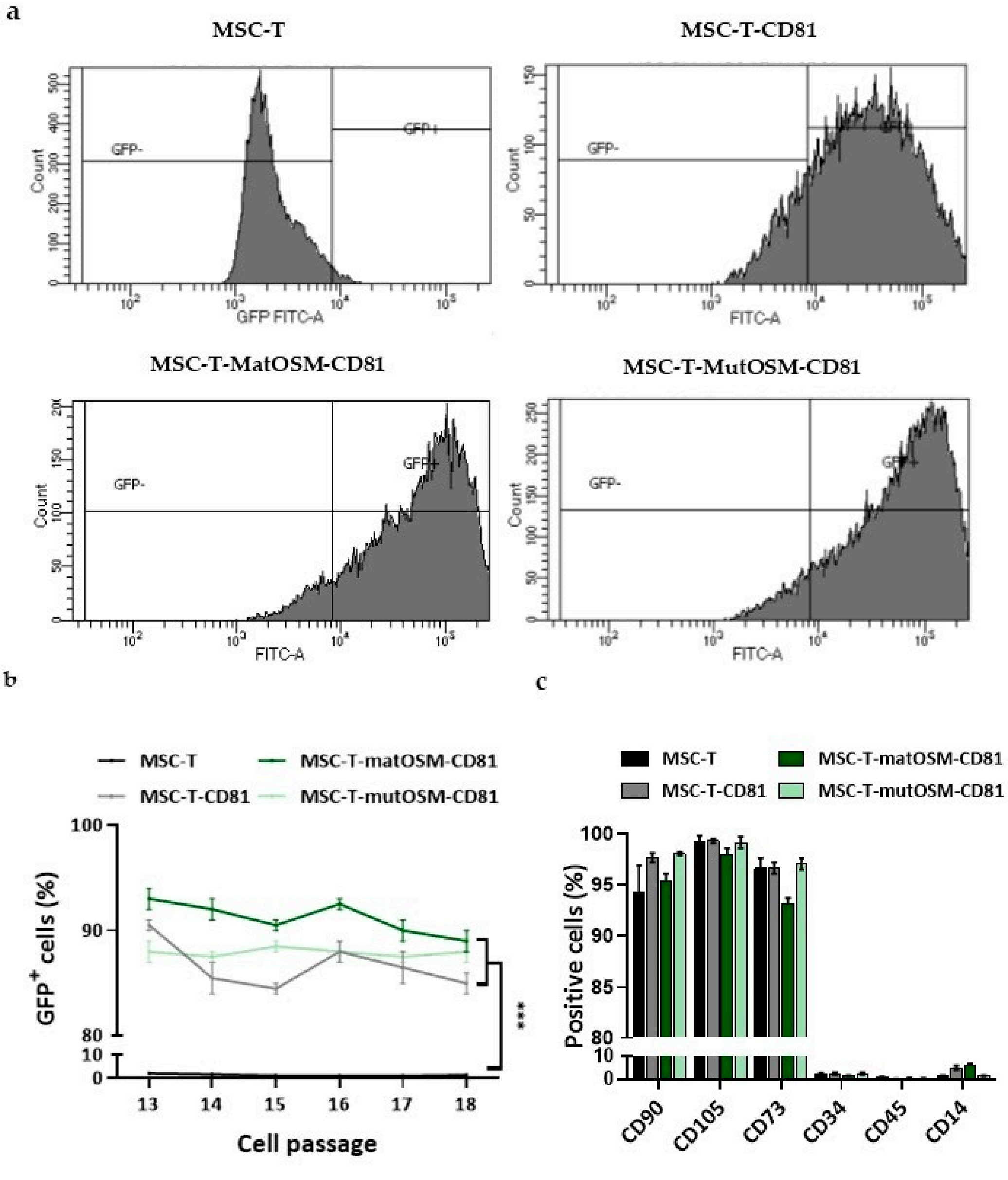

Supplementary Figure S3. Genetically modified MSC-T generation and characterization. (a) Representative histograms showing MSC-T GFP+ positive cells after CD81, matOSM-CD81 or mutOSM-CD81 plasmids introduction compared to control MSC-T. **(b)** Percentage of GFP+ cells over cell passages for MSC-T and genetically modified MSC-T measured by flow cytometry. **(c)** MSC-T and genetically modified MSC-T markers profile measured by flow cytometry. Percentage of positive cells for CD90, CD105, CD73, CD34, CD45 and CD14 on each cell type is shown. Fusion of OSM with CD81 is represented with a dash (-). Data corresponds to three independent experiments. Graphs show mean±SEM. Two-way ANOVA statistical test was used to compare data obtained from MSC-T with data obtained from MSC-T-CD81, MSC-T-matOSM-CD81 and MSC-T-mutOSM-CD81 (***$p<0.001$).

Tejedor-Gascón *et al*. Supplemental Figure S4.

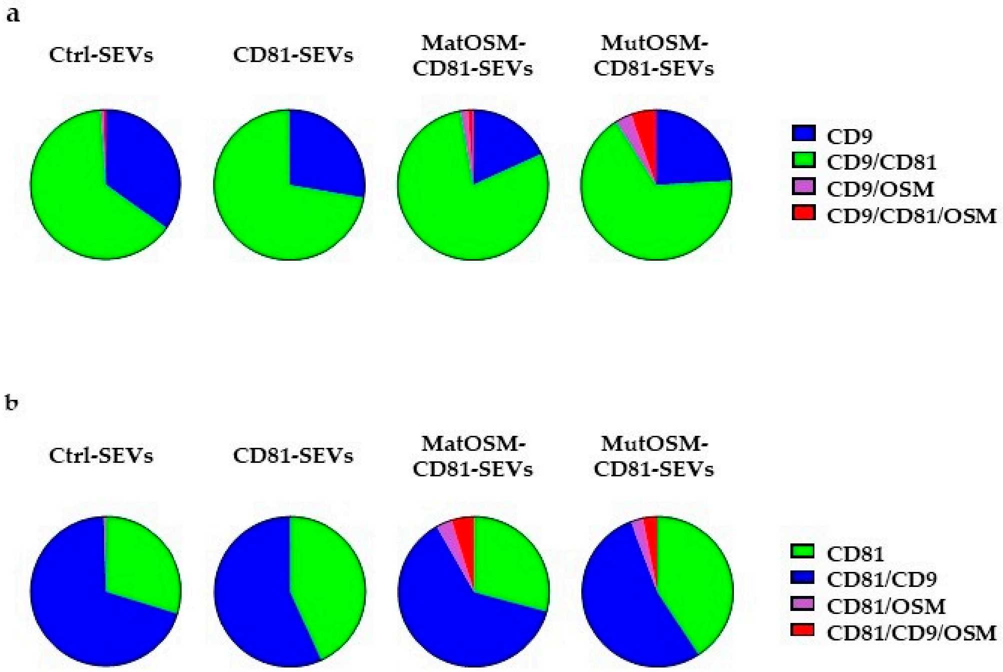

Supplementary Figure S4. Exoview colocalization analysis. (a) Pie chart of CD9 captured SEVs labeled with CD9 only or colocalized with CD81 and/or OSM. **(b)** Pie chart of CD81 captured SEVs labeled with CD81 only or colocalized with CD9 and/or OSM.
